# Different Forms of TFF3 in the Human Saliva: Heterodimerization with IgG Fc Binding Protein (FCGBP)

**DOI:** 10.3390/ijms20205000

**Published:** 2019-10-10

**Authors:** Till Houben, Sönke Harder, Harmut Schlüter, Hubert Kalbacher, Werner Hoffmann

**Affiliations:** 1 Institute of Molecular Biology and Medicinal Chemistry, Otto-von-Guericke University Magdeburg, Leipziger Str. 44, 39120 Magdeburg, Germany; till.houben@med.ovgu.de; 2 Institute of Clinical Chemistry and Laboratory Medicine, University Medical Center Hamburg-Eppendorf, Martinistr. 52, 20246 Hamburg, Germany; 3 Interfaculty Institute of Biochemistry, Eberhard-Karls-University Tübingen, Hoppe-Seyler-Str. 4, 72076 Tübingen, Germany

**Keywords:** TFF3, trefoil factor, lectin, IgG Fc binding protein, FCGBP, mucin, saliva, DMBT1

## Abstract

The peptide TFF3 is a member of a family of secretory lectins, and is typically synthesized by mucous epithelia together with mucins. It is mainly released from intestinal goblet cells as a high-molecular mass heterodimer with IgG Fc binding protein (FCGBP). Herein, we investigated human saliva by fast protein liquid chromatography (FPLC) and proteomics and identified high- and low-molecular-mass forms of TFF3. Whereas the high-molecular-mass forms represent a heterodimer with FCGBP, the low-molecular-mass forms represent homodimeric TFF3 forms. Proteomic analysis also revealed a C-terminally truncated form of TFF3. We hypothesize that salivary TFF3-FCGBP might play a role in the innate immune defense of the oral cavity and that TFF3 might also bind to microbial glycans. The known interaction of TFF3 with the agglutinin DMBT-1, a typical constituent of human saliva, further supports this protective role.

## 1. Introduction

The oral cavity is not only the entrance for food, but also a variety of microorganisms. It is protected by the oral epithelial immune barrier [1]. Here, the initial steps of alimentation are greatly facilitated by saliva. This fluid shows major individual differences and is secreted by three pairs of major glands (i.e., sublingual, submandibular, and parotid glands) and various minor glands (e.g., labial and palatal glands). Saliva fulfills a key role for the protection of teeth and the oral cavity, and is also part of the first steps of digestion [2,3,4,5]. Saliva contains a huge variety of peptides and proteins, such as enzymes, protease inhibitors, antimicrobial peptides, growth factors, mucins (MUC5B, MUC7, and MUC19), the agglutinin DMBT1, immunoglobulins (mainly secretory IgA and IgG), and surfactant proteins [5,6,7]. Saliva proteomics identified more than 3000 different proteins and their relative abundance spans 14 orders of magnitude [8]. Furthermore, also more than 600 taxa of oral microbiota secrete, e.g., enzymes, into the saliva. The major protective functions of saliva focus on both the defense against microorganisms and the support of healing processes in the oral epithelium.

In the past, members of the trefoil factor family (TFF) have also been identified in saliva. TFF peptides, i.e., TFF1-3, represent a family of secretory lectins [9,10]. TFF3 is particularly easy to detect [11]. Expression of TFFs occurs in the mucous acini of labial and submandibular glands (TFF1 at a low level, TFF2 only occasionally, and TFF3 abundantly) [11,12,13,14]. TFF3 is also detectable in parotid ducts [15]. Furthermore, TFF3 is expressed in the oral epithelium and the gingiva [13,16,17,18]. Of note, TFF3 is decreased in patients with chronic periodontitis, oral lichen planus, obstructive sleep apnea, and rhonchopathy [17,18,19].

TFF3 (formerly intestinal trefoil factor, hP1.B) consists of 59 amino acid residues, including seven cysteine residues that allow homodimerization [20]. It is typically secreted by mucous epithelia together with mucins [9,21,22]. The major expression site of TFF3 is in intestinal goblet cells, where it is released mainly as a high-molecular-mass heterodimer together with IgG Fc binding protein (FCGBP) and low amounts of TFF3 monomer and TFF3 homodimer [20,23]. TFF3 is involved in mucosal protection and repair [10,21,22], which is particularly important in the oral cavity [24]. TFFs are known to support the rapid repair of lesions by enhancing cell migration (a process called “restitution”) via their relatively weak chemotactic and anti-apoptotic activities [9]. They also have a synergistic effect with epidermal growth factor (EGF) [25,26,27]. Of note, TFF3 also enhances restitution of oral keratinocytes, which can be additionally stimulated by salivary EGF [28,29,30]. The TFF3-induced cell migration can be mediated by CXCR4 and CXCR7 [31]. However, the precise molecular mechanism is not known currently. A now favored hypothesis is that the interaction of CXCR4 and CXCR7 is via the carbohydrate moiety of these receptors [10]. This hypothesis is based upon the relatively high TFF3 concentration needed (about 10^−6^ M) and the known lectin activity of TFF3, e.g., for glycans of *Helicobacter pylori* [32,33]. In the past, TFFs were therapeutically applied to prevent mucositis, and in particular oral mucositis, in patients receiving radiation or chemotherapy [34,35,36]. Furthermore, a commercial mucin preparation sold as artificial saliva used after radiation or chemotherapy was recently shown to contain relative high amounts of TFF2 [37].

*Tff3*-deficient (*Tff3^KO^*) mice showed strongly increased sensitivity in the dextran sodium sulfate (DSS) colitis model and were particularly sensitive to chemotherapy and radiation-induced mucosal injury [21,38]. The colonic mucus is composed of two layers: a firmly attached inner and a loose outer layer. Conversion from the firm to the loose layer is likely due to a proteolytic cleavage within the gel-forming mucin MUC2 [39]. The inner layer is almost completely free of bacteria whereas the outer layer serves as the habitat and partial food source for commensal microbiota [39]. After DSS induction of colitis, the inner layer is significantly thinner and bacterial infiltration of the mucosa is lightened [40,41]. Of note, in a rat model of DSS-induced colitis, the expression of TFF3 and FCGBP were strongly reduced [42]. Based on the phenotype of *Tff3^KO^* mice, it can expected that intestinal TFF3 plays a crucial role in inhibiting bacterial infiltration through the mucus layer and thus has to be considered as part of the innate immune system. It might also play a role in autophagy [43].

Based on our previous studies of human intestinal TFF3, where we identified a TFF3-FCGBP heterodimer [23], we now investigate TFF3 in human saliva by using size exclusion chromatography (SEC) and characterized different molecular forms. This is a further step towards finally understanding the molecular function of TFF3 in saliva.

## 2. Results

### 2.1. Characterization of TFF3 in Human Saliva by SEC and Western Blot Analysis

**Figure 1:**
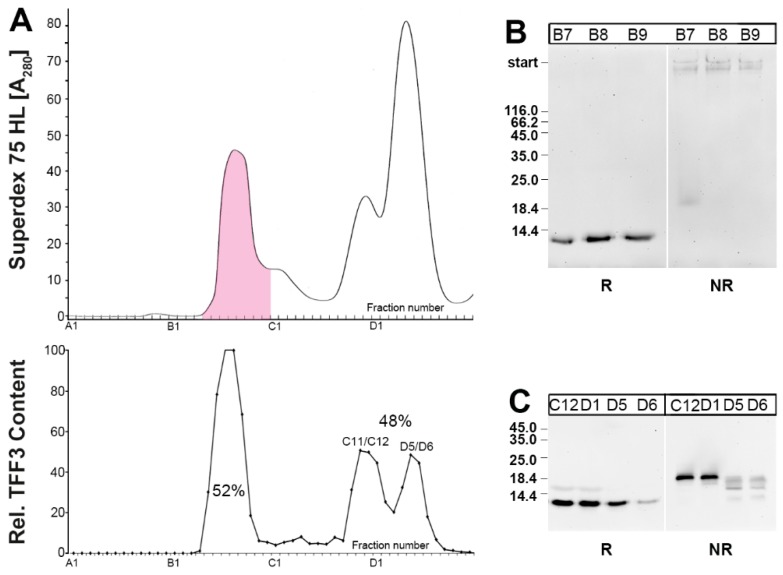
Analysis of human saliva from a single individual (S-23). (**A**) Elution profile after SEC on a Superdex 75 HL column as determined by absorbance at 280 nm. Fractions positive after PAS staining are shown in pink. Underneath: Distribution of the relative TFF3 content in the fractions as determined by Western blot analysis under reducing conditions and semi-quantitative analysis of the typical 7k-band intensities (TFF3 monomer). (**B**) 15% SDS-PAGE and subsequent Western blot analysis of the high-molecular-mass fractions B7-B9. Samples were analyzed under reducing (R) and non-reducing conditions (NR), respectively, for their TFF3 immunoreactivity. The molecular mass standard is indicated on the left. (**C**) 15% SDS-PAGE and subsequent Western blot analysis of low-molecular-mass fractions C12/D1 and D5/D6. Samples were analyzed under reducing (R) and non-reducing conditions (NR), respectively, for their TFF3 immunoreactivity. The molecular mass standard is indicated on the left.

When human saliva was separated by SEC, TFF3 immunoreactivity appeared in both a high- and low-molecular-mass range (Figure 1). TFF3 immunoreactivity in the low-molecular mass range was often split into two peaks. After reduction and SDS-PAGE, a 7k-band appeared typical of monomeric TFF3 (Figure 1B,C) [23]. Under non-reducing conditions, TFF3 immunoreactivity of the high-molecular-mass peak was greatly diminished and appeared in the high-molecular-mass range, indicating that TFF3 exists here as a heterodimer (Figure 1B).

In contrast, TFF3 immunoreactivity in the low-molecular-mass-range appeared under non-reducing conditions mainly as an 18k-band, which is typical of the TFF3 homodimer (Figure 1C). There was a difference between the two low-molecular-mass peaks, particular under non-reducing conditions, i.e., TFF3 in the C11/C12 peak contained mainly the characteristic 18k homodimeric TFF3 band, whereas TFF3 in the D5/D6 peak also appeared in somewhat smaller additional bands (Figure 1C).

**Figure 2:**
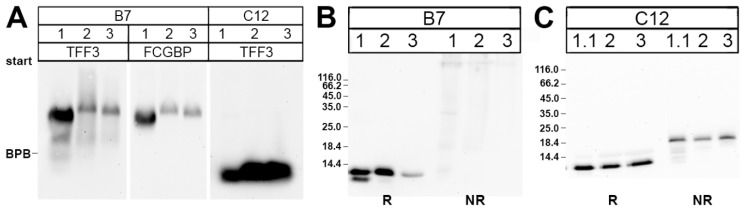
Analysis of high- and low-molecular-massfractions of human saliva of three individuals after SEC on Superdex 75 HL column. (**A**) 1% AgGE and subsequent Western blot analysis of the high- (B7) and low-molecular-mass fractions (C12), respectively, of the individuals 1–3. Shown are the reactivities for TFF3 and FCGBP, respectively. The dye bromophenol blue (BPB) is marked on the left. (**B**) 15% SDS-PAGE and subsequent Western blot analysis of the high-molecular-mass fractions B7 (after SEC on a Superdex 75 HL column) of the same three individuals as in (A). Samples were analyzed under reducing (R) and non-reducing conditions (NR), respectively, for their TFF3 immunoreactivity. The molecular mass standard is indicated on the left. (**C**) 15% SDS-PAGE and subsequent Western blot analysis of the low-molecular-mass fractions C12 analogous to (B).

In the past, the high-molecular-mass form of TFF3 from the human colon has been demonstrated to represent a disulfide-linked heterodimer with FCGBP [23]. Thus, we tested whether the high-molecular-mass form of TFF3 from human saliva also exists as a TFF3-FCGBP heterodimer. Saliva samples from three different individuals were separated by SEC on a Superdex 75 HL column and the high-molecular-mass fractions (B7) were analyzed by Western blots after AgGE (Figure 2). Clearly, in all three individual specific antisera against TFF3 and FCGBP, respectively, recognized the same bands (Figure 2A). This indicates that the high-molecular-mass form of TFF3 from human saliva is indeed a TFF3-FCGBP heterodimer. After SDS-PAGE under non-reducing conditions, TFF3 immunoreactivity was strongly diminished when compared with monomeric TFF3 and appeared in the high-molecular-mass range (Figure 2B).

In contrast, the low-molecular-mass fractions C12 did not contain TFF3-FCGBP (Figure 2A). Here, after SDS-PAGE under non-reducing conditions TFF3 immunoreactivity appeared mainly as an 18k-band characteristic of homodimeric TFF3 (Figure 2C).

### 2.2. Purification of the Low-Molecular-Mass Form of TFF3 and Characterization by Mass Spectrometric Proteomics

**Figure 3:**
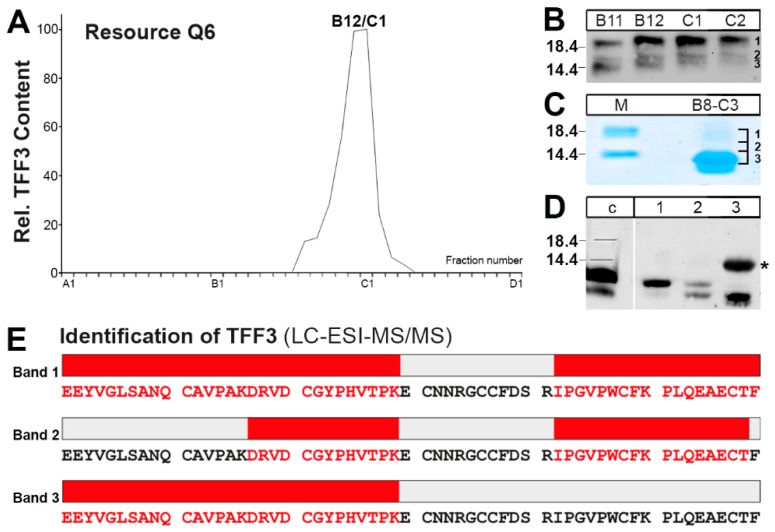
Purification of different forms of TFF3 from saliva and characterization by LC-ESI-MS/MS analysis. (**A**) Saliva of a single individual (S-20) was purified via SEC on a Superdex 75HL column (analogous to Figure 1) and then the low-molecular-mass region (about fractions C8-D5) subsequently separated by anion exchange chromatography on a Resource Q6 column. Shown is the distribution of the relative TFF3 immunoreactivity in the fractions as determined by Western blot analysis under reducing conditions and semi-quantitative analysis of the typical 7k-band intensities (monomeric TFF3). Fractions B8-C3 were concentrated, desalted and further analyzed. (**B**) 15% SDS-PAGE under non-reducing conditions of fractions B11–C2 (see (**A**)) and subsequent Western blot analysis concerning TFF3 (marked are bands 1-3). (**C**) Separation of combined fractions B8-C3 (see (**A**)) by non-reducing 15% SDS-PAGE followed by Coomassie staining. Marked are the bands excised (1, 2, 3) and subjected to Western blot analysis under reducing conditions or LC-ESI-MS/MS analysis. (**D**) Separation of the excised bands 1, 2, and 3 (see (**C**)) by 15% SDS-PAGE under reducing conditions and Western blot analysis concerning TFF3. For comparison, a human colon extract is shown (lane c). The star marks a non-specific band in lane 3 recognized by the anti-TFF3 antiserum. (**E**) Results of the LC-ESI-MS/MS analysis after tryptic in-gel digestion of the bands 1, 2, and 3, respectively. Identified tryptic peptides belonging to TFF3 are highlighted in red.

The TFF3 immunoreactive low-molecular-mass fractions from human saliva were isolated via SEC on a Superdex 75 HL column and subsequently purified by anion exchange chromatography (Figure 3A). The TFF3 peak mainly consisted of the 18k TFF3 homodimer (band 1) as well as two smaller bands 2 and 3 (Figure 3B). In order to test whether all three bands really contained TFF3, the bands were separated on a non-reducing SDS-PAGE, excised (Figure 3C), eluted, and analyzed by Western blotting on a reducing SDS-PAGE (Figure 3D). Clearly, all three bands contained TFF3 immunoreactivity, but bands 2 and 3 also contained a somewhat smaller TFF3 entity.

Furthermore, bands 1–3 were also subjected to tryptic digestion followed by LC-ESI-MS/MS analysis for protein identification (Figure 3E). Human TFF3 was identified in all three bands and, in particular, band 2 contained a TFF3 form truncated at the C-terminus by a missing phenylalanine residue.

## 3. Discussion

Here, we show for the first time that TFF3 in human saliva appears in both high- and low-molecular-mass forms. The proportion of these forms between different individuals varies, with the high-molecular-mass form ranging from about 20% to 80% (mean about 50%; data not shown). There are also variations within a single individual when compared at different time points (data not shown).

### 3.1. Salivary TFF3 Forms High-Molecular-Mass Heterodimers with FCGBP

The high-molecular-mass form of TFF3 comprises of a TFF3-FCGBP heterodimer, as has been similarly described in the human colon [23]. FCGBP contains 435 cysteine residues. This odd number would favor an intermolecular disulfide bridge, particularly with TFF3 within repeats R1, R12, and R13s [23]. In the past, FCGBP was already characterized as a constituent of human saliva [44]. Here, it is secreted from mucous acini of submandibular and labial glands together with TFF3 [14]. Thus, formation of TFF3-FCGBP heterodimers is not a solitary characteristic of the intestine. Rather, it can be expected that major proportions of TFF3 are also secreted as a TFF3-FCGBP heterodimer in other mucous epithelia, such as in the lungs and cervix uteri, because FCGBP is generally secreted by mucin producing cells [45].

Of note, reduced TFF3 monomer and heterodimeric TFF3-FCGBP exhibit clear differences in their immunoreactivities (see Figure 1 and Reference [23]). Thus, reports measuring TFF3 concentrations via enzyme-linked immunosorbent assay (ELISA) have to be taken with extreme precaution. It is absolutely essential to determine in which molecular forms TFF3 appears in the samples.

Thus far, the biological function of TFF3-FCGBP is not known. Besides binding to salivary IgG, FCGBP is generally expected to be part of a net-like scaffold within mucosal barriers [46,47]. Thus, it is a component of the first line of innate immunological defense. For example, it was hypothesized that FCGBP functions as a viral trap for HIV-1-antibody complexes at mucosal surfaces [47]. Furthermore, one might also expect that this large glycoprotein complex is also involved in an Fc-mediated killing and clearance of pathogenic bacteria. Of note, FCGBP is the highest upregulated early defense gene in catfish skin after microbial infection [48]. It is considered as a first line responder with a clear role in innate immunity critically regulating pathogen attachment and disease progression in mucosal surfaces [48]. Moreover, the heterodimerization with TFF3 could synergistically support the bacterial binding activity of FCGBP. For example, a lectin activity has been reported for TFF3 enabling binding to the lipopolysaccharide of *H. pylori* [32,33]. Thus, TFF3 as a soluble lectin could also bind to other bacterial glycans, thus exhibiting potential antibacterial activities. Recognition of microbial glycans by soluble human lectins, such as the intelectin-1 (also a constituent of human saliva [44]), is a well-known part of innate immunity defense [49]. TFF3-FCGBP could also interact with the agglutinin DMBT-1, a typical constituent of human saliva [44], because TFF3 binds to DMBT-1 [50]. This might be another indication that TFF3 is involved in microbial defense in the oral cavity since DMBT-1 aggregates microorganisms such as *Streptococcus mutans* and *S. sanguis* [3].

### 3.2. TFF3 Homodimers and Degradation in Human Saliva

The low-molecular-mass forms of TFF3 mainly consisted of the 18k-band, which is characteristic of a TFF3 homodimer [23]. Furthermore, additional TFF3 entities were detectable after SDS-PAGE under non-reducing conditions (Figure 1C and Figure 3D). All entities (bands 1–3) contained TFF3 as shown by elution from the gel and verification by reducing SDS-PAGE (Figure 3D) or by LC-ESI-MS/MS analysis (Figure 3E). The shortened variant of TFF3 in band 2 (at least lacking the C-terminal phenylalanine residue) is an indication that salivary TFF3 homodimer is subject to degradation. This could result by pepsin or bacterial proteases from the oral microbiome. For example, about 22% of healthy volunteers contained detectable levels of salivary pepsin/pepsinogen [51]. Of note, when saliva was incubated at 37 °C, we could observe degradation particularly of the TFF3 homodimer within 24h (data not illustrated). Furthermore, a shortened form of TFF3 is also detectable in the intestine (Figure 3D and Reference [23]).

The precise biological role of salivary TFF3 homodimer is not known thus far, but a protective function for the oral cavity as well as the esophagus can be expected [52]. For example, a wound healing activity is likely because TFF3 homodimer has a weak chemotactic activity. A synergistic effect with EGF (a typical constituent of human saliva) has even been described for TFF peptides [25,27,53].

Taken together, the two forms of salivary TFF3 might fulfill different protective roles for the oral cavity and esophagus. This might be of relevance for the development of new formulas for artificial saliva [54].

## 4. Materials and Methods

### 4.1. Human Saliva

All investigations followed the tenets of the declaration of Helsinki and were approved by the Ethic Committee of the Medical Faculty of the Otto-von-Guericke-University Magdeburg (code: 51/99 date: April 1999 and April 2018). Saliva samples were collected from healthy volunteers, cooled on ice, and stabilized by adding a protease inhibitor mix (Complete, EDTA-free; Roche, Mannheim, Germany; 1 tablet dissolved in 2 mL water and 50 µL were added per 1 mL saliva). The samples were then stored at −80 °C.

### 4.2. Protein Purification by FPLC

Saliva samples were centrifuged and 10 mL of the clear supernatant were fractioned by SEC with the ÄKTA^TM^-FPLC system (Amersham Biosciences, Freiburg, Germany) as described previously (fraction numbering: A1–A12, B1–B12, etc.) [37,55]. Alternatively, anion-exchange chromatography was performed as reported previously [23,37]. The following columns were used: HiLoad 16/600 Superdex 75 prep grade (S75HL; 20 mM Tris-HCl pH 7.0, 30 mM NaCl plus protease inhibitors; flow rate: 1.0 mL/min; 2.0 mL fractions) or Resource Q6 (Amersham Biosciences; salt gradient from 20 mM Tris-HCl pH 7.0 to 20 mM Tris-HCl pH 7.0 + 1 M NaCl; flow rate: 6.0 mL/min, 1.0 mL fractions).

### 4.3. SDS-PAGE, Agarose Gel Electrophoresis, and Western Blot Analysis

Non-denaturing agarose gel electrophoresis (AgGE; containing 0.1% SDS), denaturing SDS-PAGE under reducing or non-reducing conditions, protein staining with Bio-Safe Coomassie Stain G-250 (Bio-Rad Laboratories GmbH, Munich, Germany) without fixation, and periodic acid-Schiff (PAS) staining for mucins (dot blot) were described previously [23,55]. Western blot analysis after SDS-PAGE or AgGE was as reported [37].

TFF3 was analyzed with the affinity-purified polyclonal rabbit antisera anti-hTFF3-3 [23] or anti-hTFF3-8. The latter was generated against the synthetic peptide FKPLQEAECTF representing the C-terminus of human TFF3 analogous as described previously (coupling of the peptide to keyhole limpet hemocyanin was with m-maleimidobenzoyl-N-hydroxysuccinimid ester) [56]. Furthermore, a commercial polyclonal antiserum (PAP389Hu01, Cloud-Clone Corp., Katy, TX, USA) against amino acids 5176-5344 of the human FCGPB sequence was used. Bands were visualized with the ECL detection system and semi-quantitative analysis was performed using the GeneTools software (Syngene, Cambridge, UK) as described [57].

### 4.4. Mass Spectrometric Proteomics of in-Gel Digested Proteins, Database Searching

Liquid chromatography coupled to electrospray ionization and tandem mass spectrometry (LC-ESI-MS/MS) analysis of in-gel digested proteins was performed as previously described [37].

## Abbreviations

AgGE	Agarose gel electrophoresis
DMBT	Deleted in Malignant Brain Tumor
FCGBP	IgG Fc binding protein
FPLC	Fast protein liquid chromatography
LC-ESI-MS/MS	Liquid chromatography-electrospray ionization-tandem mass spectrometry
PAS	Periodic acid-Schiff
SDS-PAGE	Sodium dodecyl sulfate polyacrylamide gel electrophoresis
SEC	Size exclusion chromatography
TFF	Trefoil factor family
